# Prevalence of foot ulcers in diabetic patients in Punjab, Pakistan

**DOI:** 10.3389/fpubh.2022.967733

**Published:** 2022-08-08

**Authors:** Sohail Akhtar, Mujahid Latif, Omer Shabbir Ahmed, Aqsa Sarwar, Ayisham Alina, Muhammad Imran Khan

**Affiliations:** ^1^Department of Mathematics and Statistics, The University of Haripur, Haripur, Pakistan; ^2^Prince Sultan University, Riyadh, Saudi Arabia; ^3^Department Statistics, Government College University Lahore, Lahore, Pakistan; ^4^Department of Epidemiology and Biostatistics, School of Public Health, Anhui Medical University, Hefei, China

**Keywords:** foot ulcer, Punjab, diabetes, prevalence, Pakistan

## Abstract

Diabetes-related foot ulceration is prevalent and disabling, usually resulting in the amputation of the limb. The mortality rate is significant, and healed ulcers frequently reoccur. The main purpose of this study was to explore the prevalence of foot ulcers and their associated factors among diabetic patients in Punjab, Pakistan. Multistage cluster random sampling procedure was applied to perform a cross-sectional analysis in the state of Punjab, Pakistan. A sample of 1,503 people with diabetes, including 504 men and 999 women, were selected from different clusters. Data were collected from December 18, 2018, to June 30, 2019. Individuals of 18 years or above were selected. A binary multiple logistic regression analysis was utilized to find the factors associated with a diabetic foot ulcer. The overall prevalence of diabetic foot ulcers was 16.83% (95% CI: 14.9–18.7%). The prevalence among the female was 17.52% (95% CI: 15.2–19.9%), and the male was 15.48% (95% CI: 12.3–18.6%). In rural areas, prevalence was 13.91% (95% CI: 10.6–17.2%) compared to the prevalence of 17.96% (95% CI: 15.7–20.2%) in the urban area. Individuals 75 years and above had the highest prevalence of 66.67% (95% CI: 51.9–81.5%). According to the income status, subjects with monthly income above Rs. 61,000 had a prevalence of 24.24% (95% CI: 15.8–32.7%), and among overweight subjects was 25.49% (95% CI: 21.3–29.7%). This study found a relatively high prevalence of foot ulcers in Punjab, Pakistan. The results indicate that diabetic foot ulcers have become a major health problem in diabetic patients, and better strategies and preventive measures should be opted to deal with the epidemic.

## Introduction

Diabetes mellitus and its complications are major public health problems worldwide, affecting both developing and developed countries and costing billions of dollars in 2019; the estimated expenditure on diabetes was USD 760 billion (1). Diabetes-related lower-extremity complications significantly burden patients and society, ranking 10th among the top causes of worldwide disease burden and disability (2). A diabetic foot ulcer is one of the most serious, complex, and costly complications of diabetes. The prevalence of diabetic foot has elevated due to the worldwide increased prevalence of diabetes and the high life expectancy of diabetic patients. Globally, it is assessed that foot ulcer develops annually in 9.1–26.1 million people with diabetes (3). Between 19 and 34% of people with diabetes will develop a foot ulcer at some point in their lives (4). Diabetes and its related complications account for 12% of global health expenditure of total health care expenditure. The chance of demise for a patient with a diabetic foot ulcer at 5 years is 2.5-fold higher compared to patients with no foot ulcer (5). Over the recent decades, the prevalence of diabetes has drastically increased all over the world. International Diabetes Federation (IDF) estimated that in 2019 around 463 million people had diabetes, and by 2045 this number is expected to rise by 700 million (6). It is also reported that diabetes will increase by 69% in under-developing nations and 20% in developed countries between 2010 and 2030 (7).

Foot ulcers and amputations are common among diabetic patients in Pakistan. This is due to the lack of adequate health care, poverty, and a lack of proper sanitation and hygiene (7–9). People with diabetes reported a negative impact on foot health-related quality of life, which appears to be connected to the prevalence of chronic disease in the diabetes community (10). Comparing patients with type I and type II diabetes, patients with type II diabetes have a negative influence on foot-related quality of life (11). As foot ulcer is among the most expensive complications of diabetes, it increases a substantial burden on individuals and society in the form of higher medical expenditure, lost productivity, and premature mortality (12). With the limited health facilities for diabetes and its related complications, Pakistan is ill-equipped to manage this epidemic. Several studies have been conducted to investigate the prevalence of foot ulcers and assess the factors related to foot ulcers among people with diabetes in Pakistan (13–17). These investigations showed that a patient's age, educational level, weight, type of diabetes mellitus, foot self-care routines, and presence of severe peripheral neuropathy all have an impact on developing a diabetic foot ulcer. However, there are differences in the socioeconomic and demographic characteristics that influence diabetic foot ulcers. In order to minimize the fatal effects of foot ulcers among diabetes patients, it is crucial to examine factors impacting diabetic foot ulcers in many areas. Therefore, the purpose of the present study was to investigate the prevalence of diabetic foot ulcers and associated risk factors among diabetic patients in Punjab, Pakistan. The results of this study will contribute to a reduction in the frequency of diabetic foot ulcers and their complications in the region.

## Methods

### Study population, study design, and sampling method

A cross-sectional analysis was carried out from December 2018 to June 2019. The individuals having an age of 18 or above were selected from the target population. The multistage cluster random sampling method was used to recruit subjects to collect the representative sample from Punjab. Punjab is the most inhabited province of Pakistan, and the total population is 110,012,442 (16). There are 36 districts in Punjab, each marked as a single cluster (17). At the first sampling stage, two random districts (Lahore and Kasur) were selected from all Punjab districts (clusters). At the second stage of sampling, ten union councils (sub-clusters) were chosen out of 150 union councils of district Lahore. In comparison, seven union councils (sub-cluster) were chosen out of 113 union councils of district Kasur (18). In the third sampling stage, each selected union council was subdivided into different subclusters (streets). Using systematic random sampling, every 15th street was visited for data collection, and only diabetic patients (who were already confirmed by physicians) from every household were considered for collecting information. A total of 1,503 Individuals (male and female) were selected from those selected streets.

### Sample size calculation

The sample size was calculated by employing prior information about the prevalence of foot ulcers of 14.7% among patients of Punjab having diabetes. A 95% confidence level, ±1.79% precision, and 80% study power resulted in a sample size of 1,503 participants (19).

### Data collection

A pretested questionnaire was used for a face-to-face interview of each patient, and informed written approval was obtained before the interview. The questionnaire was pretested using a pilot survey. Questions about patients' socio-demographic and living characteristics that include age, area of residence, gender, education, marital status, profession and monthly family earnings, BMI, smoking status, and physical activity were included in the first part of the questionnaire. The following part of the questionnaire was designed to collect clinical and diabetes-related evidence from the participants, such as duration of diabetes, hypertension, family history of foot ulcer, and neuropathy symptoms. A data extracted checklist was used to collect statistics from patients' records, including laboratory test results, diagnosis, and past medical history.

### Ethics approval

Government College University's Review Board investigated the study and provided ethical approval (Reference No. GCU-IIB-229, dated 10-10-2018) for further research. In the study involving human beings, all actions were according to the ethical standards of the institutional and national research committee. In addition, the questionnaire was approved by doctors and obtained informed consent from all individual participants included in the study.

### Statistical analysis

Displayed the categorical data in frequency and percentages. Quantitative data were represented as mean (and standard deviation) and median (and percentiles), where appropriate. Simple and multiple binary logistic regressions were used to identify the variables related to a diabetic foot ulcer. A <0.05 *p*-value was considered statistically significant. Statistical software Minitab version 18.0 was used to perform all data analysis. Two us authors (SA and AS) independently perform the statistical analysis to compare the reliability of the results.

## Results

Table 1 presents the demographic and socioeconomic characteristics of the survey respondents. Out of the 1,503 participants in this study, 79% had T2DM, and 66.5% were female. The overall mean age was 51.58 ± 11.491 years, and that of T1DM and T2DM were 46.20 ± 13.551 and 52.02 ± 11.313, respectively. The mean duration of diabetes was 5.652 ± 3.812 years. Only 9% of the participants smoked, and 72.3% were from urban areas.

**Table 1:** Demographic and socioeconomic characteristics of the study participants.

**Variables**	**Number (%)**	**Variables**	**Number (%)**
**Age**		**Education level**	
18–34	113 (7.5)	Illiterate	369 (24.5)
35–54	757 (50.4)	Matric	644 (42.8)
55–74	594 (39.5)	Intermediate	224 (14.9)
75+	39 (2.6)	Graduation or more	266 (17.7)
**Gender**		**Place of residence**	
Male	504 (33.5)	Urban	1,086 (72.3)
Female	999 (66.5)	Rural	417 (27.7)
**Marital Status**		**Hypertensive**	
Unmarried	60 (3.9)	Yes	591 (39.3)
Married	1,443 (96)	No	912 (60.7)
**BMI (kg/m** ^**2**^ **)**		**Smoking**	
Healthy	536 (35.7)	Non-Smoker	1,275 (84.8)
Overweight	412(27.4)	Current	132 (8.8)
Obesity	555 (36.9)	Ex-Smoker	96 (6.3)
**Income (PKR)**		**Walk (in minutes per day)**	
<20,000	759 (50.5)	No	795 (52.9)
20,000–40,000	519 (34.5)	Mild (15–29 min)	168 (11.2)
41,000–60,000	126 (8.4)	Moderate (30–59 min)	357 (23.8)
>61,000	99 (6.6)	Heavy (more than 1 h)	183 (12)

**Figure 1:**
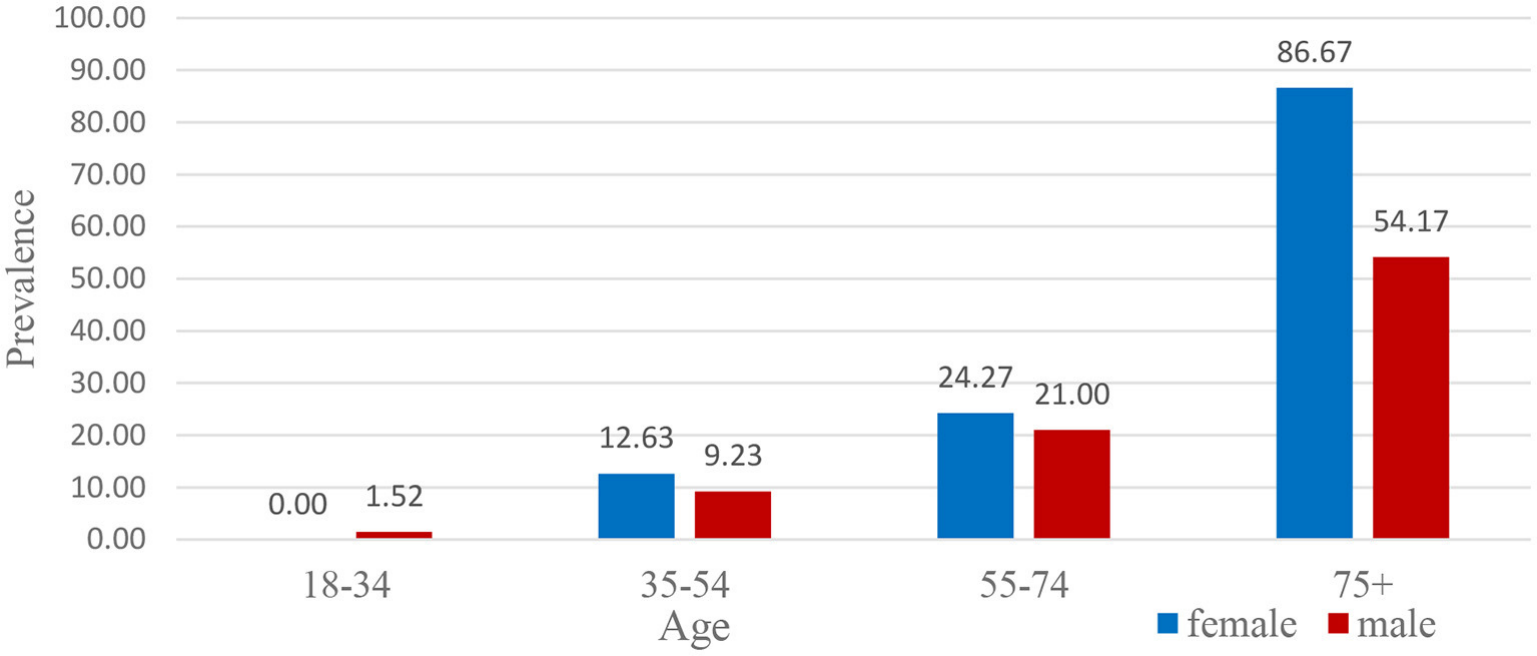
Percentage of foot ulcers in diabetic subjects according to age.

Table 2 indicates the prevalence of foot ulcers by participants' characteristics. About 16.83% (95% CI: 14.9–18.7%) of the participants had foot ulcers, and among the females, 17.52% (95% CI, 15.2–19.9%) had foot ulcers compared to 15.48% (95% CI, 12.3–18.6%) among male. Among the rural participants, 13.91% (95% CI, 10.6–17.2%) had foot ulcers, and that was 17.96% (95% CI, 15.7–20.2%) among the urban residents. The percentage of foot ulcers increased progressively as age increased and was the highest (Figure 1), 66.67% (95% CI, 51.9–81.5%), for the participants aged 75 years and above. The participants with healthy BMI had foot ulcers of 10.26% (95% CI, 7.7–12.8%) compared to 25.49% (95% CI, 21.3–29.7%) for overweight and 16.76% (95% CI, 13.6–19.9%) for obesity, their difference is statistically significant.

**Table 2:** Foot ulcer status by the study participants' characteristics.

	**Total**		**Non-Ulcer**		**Ulcer**		**Prevalence of FU**	**(95%CI)**
**Overall**	1,503		1,250		253		16.83	14.9–18.7
**Variables**	***n***	**%**	***n***	**%**	***N***	**%**	**%**	**%**
**Age**								
18–34	113	7.52	112	8.96	1	0.40	0.88	0.8–2.6
35–54	757	50.37	668	53.44	89	35.18	11.75	9.5–14.1
55–74	594	39.52	457	36.56	137	54.14	23.06	19.7–26.5
75+	39	2.59	13	1.04	26	10.28	66.66	51.9–81.5
**Gender**								
Male	504	33.53	426	34.08	78	30.83	15.48	12.3–18.6
Female	999	66.47	824	65.92	175	69.17	17.52	15.2–19.9
**Place of residence**								
Urban	1,086	72.26	891	71.28	195	77.08	17.96	15.7–20.2
Rural	417	27.74	359	28.72	58	22.92	13.91	10.6–17.2
**Income (PKR)**								
<20,000	759	50.50	630	50.40	129	50.99	17.00	14.3–19.7
20,000–40,000	519	34.53	439	35.12	80	31.62	15.41	12.3–18.5
41,000–60,000	126	8.38	106	8.48	20	7.91	15.87	9.5–22.3
>61,000	99	6.59	75	6.00	24	9.49	24.24	15.8–32.7
**BMI**								
Healthy	536	35.66	481	38.48	55	21.74	10.26	7.7–12.8
Overweight	412	27.41	307	24.56	105	41.50	25.49	21.3–29.7
Obesity	555	36.93	462	36.96	93	36.76	16.76	13.6–19.9

**Figure 2:**
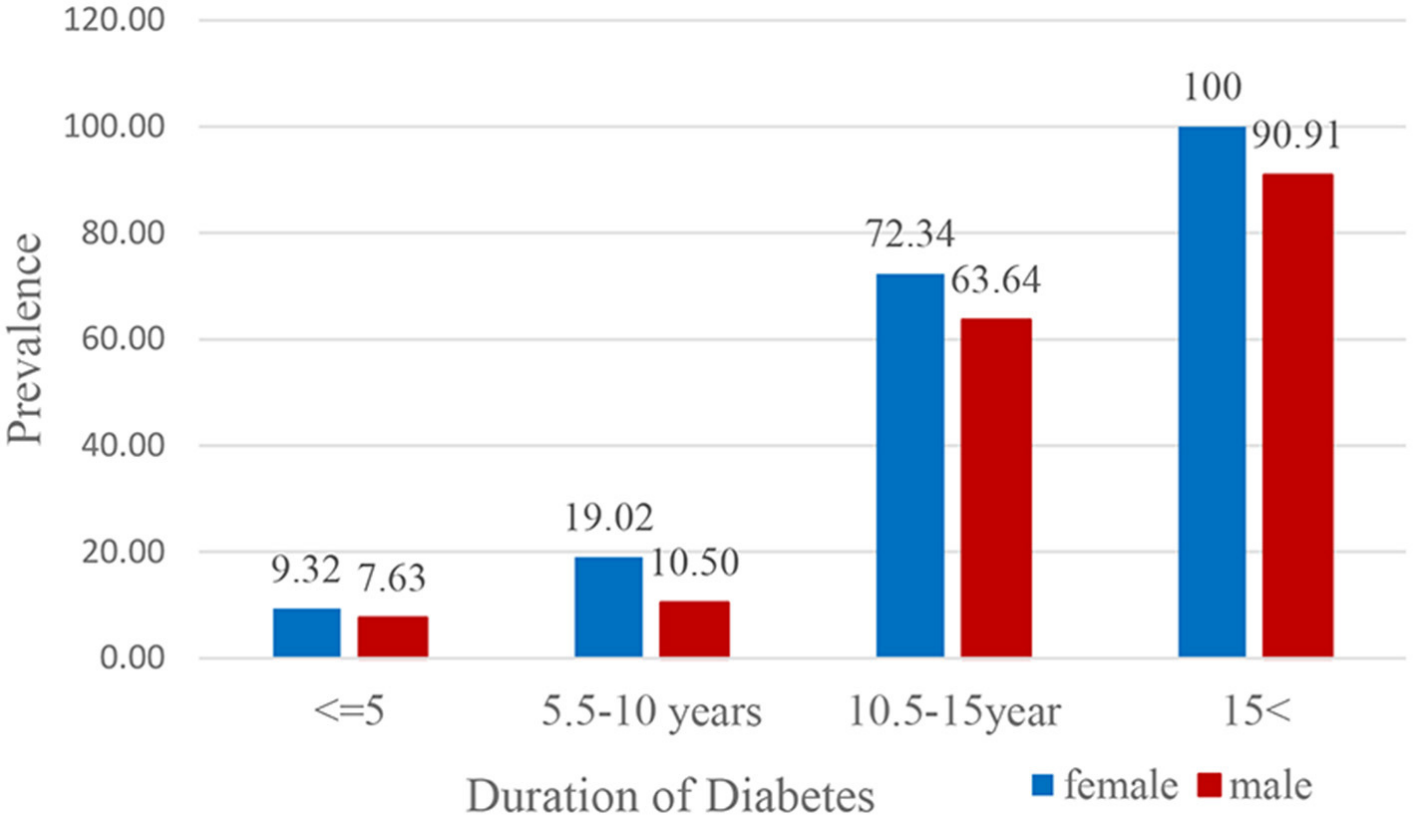
Prevalence of diabetic foot ulcer in diabetic subjects according to the duration of diabetes.

At 18–34 years old, it was observed that males had foot ulcers more than females of this age group, i.e., 1.52 and 0%, respectively. There were 9.23 and 12.63% cases of foot ulcers found in males and females aged 35–54 years. However, females 55–74 years of age were diagnosed with foot ulcers more than males, i.e., 24.27% female and 21.00% male. It was demonstrated from the graph that 54.17% of males of age 75 and above were suffering from foot ulcers, and 86.67% of females had foot ulcers in that age group. The percentage of foot ulcers in the higher age group was also very high (Figure 2). The reason was the immune system of people of this age group became very weak, and they were least resistant to any disease.

Table 3 displays the binary and multiple logistic regression analysis with Odds Ratios at a 95% confidence interval. The odds ratio is >1 describes a positive relationship. The above table shows that as the age and duration of diabetes increases, the risk of foot ulcers also increases. The odds of 1.906 and 1.212 indicate that the foot ulcer had a higher risk of amputation. The odds ratio of gender 0.418 and 0.514 were >1 implying a negative relationship. This means that being male would correspond to lower odds of having foot ulcers. The model summary table presents statistics examining how well the overall model fits the data. In this result, the model explains 35% of the deviance in the response variables.

**Table 3:** Odds Ratios, confidence interval, chi-square, and *p*-value of the variables under study.

**Variable**	**Simple logistic regression**			**Multiple logistic regression**		
	**OR**	**95% CI**	***p***	**OR**	**95% CI**	***p***
Age	1.072	(1.057–1.087)	0.000	1.072	(1.050–1.095)	0.000
Duration of diabetes	1.416	(1.3495–1.486)	0.000	1.391	(1.317–1.470)	0.000
Gender Male = 1 Female = 0	0.862	(0.644–1.154)	0.318	3.256	(1.840–5.760)	0.000
Wash feet regularly	0.419	(0.271–0.648)	0.000	0.515	(0.315–0.843)	0.005
Smoking status						
Ex-Smoker = 1 Current = 2, None = 0	1.167	(0.944–1.443)	0.154	0.925	(0.704–1.216)	0.572
Education level						
Illiterate = 1, Matric = 2, Intermediate = 3, Graduate = 4	0.665	(0.597–0.741)	0.000	0.354	(0.278–0.452)	0.000
Type of diabetes	1.292	(0.991–1.831)	0.143	1.123	(0.7340–1.720)	0.590
Amputation	1.906	(1.180–3.080)	0.012	1.212	(0.5375–2.734)	0.645
Deformity	1.345	(0.860–2.104)	0.195	0.751	(0.3601–1.569)	0.441
Time to experience ulcer	1.093	(0.988–1.208)	0.095	0.986	(0.844–1.151)	0.854

## Discussion

Ulceration of the foot in diabetic patients is mostly preventable; however, it remains a substantial cause of hospitalization in diabetic patients throughout the world. Therefore, this study aimed to investigate the prevalence of diabetic foot ulcers and their risk factors among diabetic patients in Punjab, Pakistan. The study findings showed that the prevalence of diabetic foot ulcers among diabetic patients was 16.83% (95% CI: 14.9–18.7%). The diabetic foot ulcer prevalence was lower in this study than in studies conducted in Sudan (18.1%) (20), and Nigeria (41%) (21). This discrepancy may be attributable to changes in sample size, geographical region, the age structure of the participants, and sociocultural variation among study participants.

On the other hand, the current study's findings show that the prevalence of foot ulcers is greater when compared to a study conducted in Ethiopia, which revealed that the prevalence of diabetic foot ulcerations among diabetic patients was 13.6% (22). This disparity could be explained by differences in understanding of foot self-care practice, diabetes control knowledge, and possibly health-seeking behavior practice could all account for this gap between the two study populations.

Our findings showed that the prevalence of foot ulcers in diabetic patients in the 18–34year age group was the lowest (0.88%), while the highest prevalence was reported in the 75 and older age group (66.66%). Peripheral neuropathy, foot abnormalities, and peripheral arterial disease are the most common causes of foot disease (23).

The results of this study also revealed that the prevalence of foot ulcers in overweight, diabetic patients (25.49%) was significantly higher than the normal weight patients (10.26%). This means that overweight, diabetic patients were ~2.5 times more likely to develop diabetic foot ulcers as compared normal weight diabetic patients. The findings align with the research conducted in Ethiopia, and Saudi Araba (22, 24). The presence of increased foot pressure in those who are overweight and strongly built may significantly reduce the normal blood flow pattern to the lower extremities, which may cause them to develop diabetic foot ulcers.

Our findings suggest that untreated wounds and high blood glucose levels are the reason for developing foot complications. A higher prevalence of foot ulcers among female diabetic patients than among male diabetic patients' diabetic patients has been observed. This gender difference was well-documented in previous research conducted in Pakistan (13) Error! Bookmark not defined. and other countries (25, 26). However, a study was conducted in Pakistan (27), and in another county, males outnumbered females, which contradicts our study (25). A prevalence in males might be because of the more disclosure of trifling foot injury, barefoot walking, and footwear shock.

The urbanites of Punjab had a higher rate of having foot ulcers, which is incoherent with the findings of prior studies conducted in different cultural and geographical settings across the region. People with higher income status had a higher prevalence of foot ulcers, i.e., 24.24%. It showed that people with higher income status had less physical activity. This factor was not discussed in any past study held in Punjab.

Hypertension is a significant independent risk factor for the growth of neuropathy and diabetic foot ulcers and is found in 60.68%. The percentage of deformity in diabetic subjects was 8.6%. It also leads to amputation and death. Additionally, foot ulcer-driven complications increase the expenditure on medical care. Consequently, there is an urgent need for policymakers of the country to suggest the prevention and control of diabetic foot ulcers. As this situation is disastrous for the health of the masses and the country's economy, we must tackle it on a priority basis.

The prevalence of diabetic foot ulcers in Pakistan is projected to rise as the number of persons with diabetes rises in Pakistan (26–29). Most of the world suffers from a serious dearth of foot care professionals and facilities, and Pakistan is one of the nations with limited foot care services available. The most effective strategy is proper diabetes management, but secondary prevention is the goal of good foot-ulcer care (30). Furthermore, the progression to limb amputation will be stopped through patient education regarding self-examining the foot and identifying people with diabetes are at increased risk of developing foot ulcers. Better prognosis is ensured by proper diabetic foot ulcer assessment and management. Emerging technologies may be considered for quantifying risks, prevention, and management of diabetic foot ulcers (31, 32). Furthermore, minimally invasive surgery (33–35) is one the effective procedures for treating complicated foot ulcers, which may be considered in Pakistan for the treatment of foot ulcers.

There are various limitations to our study. First, women and urban diabetic patients were oversampled, and men responded at a lower rate than women. When calculating statistical weights, we considered these difficulties. However, among men, the response rate was more than 78%, raising the possibility of sample bias due to non-responses.

## Conclusion

Our findings from this current study show that the prevalence of foot ulcers in diabetic patients in Punjab, Pakistan is relatively high and that immediate preventive measures should be implemented. Diabetic foot ulcer needs considerable efforts and dedicated actions for the prevention of the disease. Control diabetes, early detection and modification of the risk factors for the development. The importance of diabetic foot ulceration awareness programs and population-based screening campaigns should not be underestimated. These initiatives will undoubtedly assist to minimize the disease's burden. The findings will help them understand the impact of foot ulcers on diabetic patient risk factors in the area. However, more research is needed to determine the relationship between these risk factors.

## Data availability statement

The raw data supporting the conclusions of this article will be made available by the authors, without undue reservation.

## Ethics statement

The studies involving human participants were reviewed and approved by Government College University's Review Board investigated the study and provided ethical approval (Reference No. GCU-IIB-229, dated 10-10-2018). The patients/participants provided their written informed consent to participate in this study.

## Author contributions

All authors listed have made a substantial, direct, and intellectual contribution to the work and approved it for publication.

## Funding

The authors would like to acknowledge the support of Prince Sultan University for paying the Article Processing Charges (APC) of this publication.

## Conflict of interest

The authors declare that the research was conducted in the absence of any commercial or financial relationships that could be construed as a potential conflict of interest.

## Publisher's note

All claims expressed in this article are solely those of the authors and do not necessarily represent those of their affiliated organizations, or those of the publisher, the editors and the reviewers. Any product that may be evaluated in this article, or claim that may be made by its manufacturer, is not guaranteed or endorsed by the publisher.

## References

[1] Williams R Karuranga S Malanda B Saeedi P Basit A Besançon S. Global and regional estimates and projections of diabetes-related health expenditure: results from the International Diabetes Federation Diabetes Atlas. Diabetes Res Clin Pract. (2020) 162:108072. 10.1016/j.diabres.2020.108072 32061820

[2] Lazzarini P Pacella RE Armstrong D Van Netten J. Diabetes-related lower-extremity complications are a leading cause of the global burden of disability. Diabetic Med. (2018) 35:1297–9. 10.1111/dme.13680 29791033

[3] Federation, ID. IDF Diabetes Atlas 10th Edition 2021. Available online at: https://diabetesatlas.org/idfawp/resource-files/2021/07/IDF_Atlas_10th_Edition_2021.pdf (accessed April 20, 2022).

[4] Armstrong DG Boulton AJ Bus SA. Diabetic foot ulcers and their recurrence. N Engl J Med. (2017) 376:2367–75. 10.1056/NEJMra1615439 28614678

[5] Romero Prada M Roa C Alfonso P Acero G Huérfano L Vivas-Consuelo D. Cost-effectiveness analysis of the human recombinant epidermal growth factor in the management of patients with diabetic foot ulcers. Diabetic Foot Ankle. (2018) 9:1480249. 10.1080/2000625X.2018.1480249 29963295 PMC6022247

[6] Rehman R Malik FR Rehman Z. A comparative study on diabetic foot ulcers leading to amputations. J Postgrad Med Inst. (2018) 32. Available online at: https://jpmi.org.pk/index.php/jpmi/article/view/2030

[7] Hayat AS Baloch GH Bawany MA Shaikh N. Peripheral arterial disease:(Pad) in type 2 diabetic patients. Professional Med J. (2012) 19:501–7. 10.29309/TPMJ/2012.19.04.2267

[8] Khan A Junaid N. Prevalence of diabetic foot syndrome amongst population with type 2 diabetes in Pakistan in primary care settings. JPMA. (2017) 67:1818–24. https://www.jpma.org.pk/PdfDownload/8473 29256523

[9] Ansari S Memon IA Shaikh NA Memon KI. Frequency of peripheral arterial disease in diabetic foot. Jlumhs. (2009) 8. Available online at: https://www.lumhs.edu.pk/jlumhs/Vol08No03/pdfs/v8n3oa01.pdf

[10] López LL Iglesias ME Gómez-Salgado J de Bengoa Vallejo RB Morales CR López DL. The implications of diabetic foot health-related with quality of life: a retrospective case control investigation. J Tissue Viability. (2022) S0965-206X(22)00077-8. 10.1016/j.jtv.2022.07.003 35853797

[11] Palomo-López P Losa-Iglesias ME Becerro-de-Bengoa-Vallejo R López-López D Rodríguez-Sanz D Romero-Morales C. Specific foot health-related quality-of-life impairment in patients with type II versus type I diabetes. Int Wound J. (2019) 16:47–51. 10.1111/iwj.12984 30168292 PMC7948763

[12] Abdul Waheed Jan HK Ahmad Ahmad I Khan M. Diabetic foot ulcer. Prof Med J. (2016) 23:693–8. 10.17957/TPMJ/16.3288

[13] Younis BB Shahid A Arshad R Khurshid S Ahmad M Yousaf H. Frequency of foot ulcers in people with type 2 diabetes, presenting to specialist diabetes clinic at a Tertiary Care Hospital, Lahore, Pakistan. BMC Endocr Disord. (2018) 18:53. 10.1186/s12902-018-0282-y 30081878 PMC6090692

[14] Amjad SS Shams N Zahra T. Diabetic foot ulcers in a tertiary care hospital; risk factors, associations and grades of presentation. JLUMHS. (2016) 15:5–11. 10.22442/jlumhs.161510456

[15] Khan MIH Azhar U Zubair F Khan ZA. Can we link foot ulcer with risk factors in diabetics? A study in a tertiary care hospital. Pak J Med Sci. (2018) 34:1375–80. 10.12669/pjms.346.16199 30559788 PMC6290204

[16] Pakistan, Tehsil Wise For Web Census_2017,.pdf. Province Wise Provisional Results of Census - 2017. Available online at: http://www.pbs.gov.pk/sites/default/files/PAKISTAN%20TEHSIL%20WISE%20FOR%20WEB%20CENSUS_2017.pdf (accessed December 29, 2019).

[17] Punjab, Portal. Quick Stats. Pakistan Bureau of Statistics Census Results 2017. Available online at: https://punjab.gov.pk/punjab_quick_stats (accessed December 29, 2019).

[18] Pakistan Bureau of Statistics. Punjab Pakistan Bureau of Statistics. Available online at: http://www.pbs.gov.pk/pco-punjab-tables (accessed December 29, 2019).

[19] G. P. Sample Size (2013). Available online at: http://sampsize.sourceforge.net/iface/ (accessed June 20, 2020).

[20] Almobarak AO Awadalla H Osman M Ahmed MH. Prevalence of diabetic foot ulceration and associated risk factors: an old and still major public health problem in Khartoum, Sudan? Ann Transl Med. (2017) 5:340. 10.21037/atm.2017.07.01 28936434 PMC5599292

[21] Ogbera A Adedokun A Fasanmade O Ohwovoriole A Ajani M. The foot at risk in Nigerians with diabetes mellitus-the Nigerian scenario. Int J Endocrinol Metab. (2005) 3:165–73.

[22] Abdissa D Adugna T Gerema U Dereje D. Prevalence of diabetic foot ulcer and associated factors among adult diabetic patients on follow-up clinic at Jimma Medical Center, Southwest Ethiopia, 2019: an institutional-based cross-sectional study. J Diabetes Res. (2020) 2020:4106383. 10.1155/2020/4106383 32258165 PMC7102459

[23] Al-Rubeaan K Al Derwish M Ouizi S Youssef AM Subhani SN Ibrahim HM. Diabetic foot complications and their risk factors from a large retrospective cohort study. PLoS ONE. (2015) 10:e0124446. 10.1371/journal.pone.0124446 25946144 PMC4422657

[24] Musa IR Ahmed MO Sabir EI Alsheneber IF Ibrahim EM Mohamed GB. Factors associated with amputation among patients with diabetic foot ulcers in a Saudi population. BMC Res Notes. (2018) 11:260. 10.1186/s13104-018-3372-z 29703224 PMC5921536

[25] Alzahrani HA Sehlo MG. The impact of religious connectedness on health-related quality of life in patients with diabetic foot ulcers. J Relig Health. (2013) 52:840–50. 10.1007/s10943-011-9529-x 21863475 PMC3695669

[26] Akhtar S Nasir JA Abbas T Sarwar A. Diabetes in Pakistan: a systematic review and meta-analysis. Pak J Med Sci. (2019) 35:1173–8. 10.12669/pjms.35.4.194 31372163 PMC6659044

[27] Basit A Fawwad A Qureshi H Shera A. Prevalence of diabetes, pre-diabetes and associated risk factors: second National Diabetes Survey of Pakistan (NDSP), 2016–2017. BMJ Open. (2018) 8:e020961. 10.1136/bmjopen-2017-020961 30082350 PMC6078264

[28] Akhtar S Shah SWA Javed S Alina A. Prevalence of diabetes and prediabetes in district swat Pakistan. J Pak Med Assoc. (2021) 71:243–6. 10.47391/JPMA.548 35157657

[29] Akhtar S Khan Z Rafiq M Khan A. Prevalence of type II diabetes in District Dir Lower in Pakistan. Pak J Med Sci. (2016) 32:622–5. 10.12669/pjms.323.9795 27375702 PMC4928411

[30] Jeffcoate WJ Harding KG. Diabetic foot ulcers. Lancet. (2003) 361:1545–51. 10.1016/S0140-6736(03)13169-8 12737879

[31] Balasubramanian GV Chockalingam N Naemi R. The role of cutaneous microcirculatory responses in tissue injury, inflammation and repair at the foot in diabetes. Front Bioeng Biotechnol. (2021) 9:732753. 10.3389/fbioe.2021.732753 34595160 PMC8476833

[32] Lung C-W Wu F-L Liao F Pu F Fan Y Jan Y-K. Emerging technologies for the prevention and management of diabetic foot ulcers. J Tissue Viability. (2020) 29:61–8. 10.1016/j.jtv.2020.03.003 32197948

[33] Biz C Belluzzi E Crimì A Bragazzi NL Nicoletti P Mori F. Minimally Invasive Metatarsal Osteotomies (MIMOs) for the Treatment of Plantar Diabetic Forefoot Ulcers (PDFUs): a systematic review and meta-analysis with meta-regressions. Appl Sci. (2021) 11:9628. 10.3390/app11209628

[34] Biz C Gastaldo S Dalmau-Pastor M Corradin M Volpin A Ruggieri P. Minimally invasive distal metatarsal diaphyseal osteotomy (DMDO) for chronic plantar diabetic foot ulcers. Foot Ankle Int. (2018) 39:83–92. 10.1177/1071100717735640 29110516

[35] Biz C Ruggieri P. Minimally invasive surgery: osteotomies for diabetic foot disease. Foot Ankle Clin. (2020) 25:441–60. 10.1016/j.fcl.2020.05.006 32736741

